# F244. CHILDHOOD PSYCHOTIC EXPERIENCES ARE ASSOCIATED WITH PERSISTENTLY POORER FUNCTIONING INTO YOUNG ADULTHOOD: A 9-YEAR FOLLOW-UP STUDY

**DOI:** 10.1093/schbul/sby017.775

**Published:** 2018-04-01

**Authors:** Dónal Campbell, Colm Healy, Mary Cannon

**Affiliations:** Royal College of Surgeons in Irel

## Abstract

**Background:**

Psychotic experiences (PEs) are relatively common in childhood and early adolescence, being present in 17% of children aged 9 to 12 (Kelleher et al., 2012). Research suggests that young people who experience PEs are more vulnerable to psychopathology later in life, despite PEs being transient in 78.7% of cases (Zammit et al., 2013). While childhood PEs are associated with poorer functioning (Kelleher et al., 2015), it has not yet been established whether the impact of PEs on functioning persists into later life.

**Methods:**

52 participants from a prospective cohort study (retention rate: 60.4%) of Irish young people were included on the basis that they had completed a clinical interview at all three data-collection time points (T1 mean age: 11.69; T2: 15.80; T3 18.80). Following each interview, participants were scored on the Global Assessment of Functioning (GAF) scale, and given a Current (C-GAF) score and a Most Severe Past (MSP-GAF) score. Fixed-effects repeated-measures models were used to compare the scores of those with a history of PEs at T1 (n=18) to those without (n=34), accounting for age, gender, and childhood functioning. Secondary analyses investigated whether differences in functioning were evident in those who reported transient PEs (only at T1; n=12).

**Results:**

Overall, participants who had reported childhood PEs (T1) received significantly lower C-GAF scores (F = 31.553, p < .001) and MSP-GAF scores (F = 79.377, p < .001) than those without PEs. Simple effects analysis indicated that deficits in the PE group were evident at each time point for both C-GAF scores (T1: p = .001; T2: p <.001; T3: p = .002) and MSP-GAF scores (T1: p < .001; T2: p = .001; T3: p < .001), indicating poorer functioning from childhood, through adolescence, into early adulthood. There was no significant effect of the co-variates.

When the analysis was restricted to a comparison of participants who reported PEs at T1 only (i.e. transient PEs) and those with no history of PEs, the PE group had poorer functioning scoring than their peers across the three time points (C-GAF: F = 17.709, p < .001; MSP-GAF: F= 32.247, p < .001).

**Discussion:**

The analysis provides longitudinal evidence that the presentation of PEs is associated with persistent poor global functioning throughout adolescence and into early adulthood, even when the phenomena are transient. PEs appear to be a marker for vulnerability that extends beyond mental disorder. These results tentatively suggest a causal link between PEs and poorer functioning later in life, as the difference in functioning between the groups in early adulthood was still evident after accounting for childhood functioning. Moreover, the disparity between the groups is clinically relevant, with the PE group scoring one to two categories lower than their peers on the GAF scale even into early adulthood. Childhood PEs are an excellent prognostic marker for future functioning and providing targeted early intervention for these individuals may reduce the likelihood of developing a significant clinical disorder later in life.

